# Supplementary material for: Evidence and Future Perspectives for Neoadjuvant Therapy for Resectable and Borderline Resectable Pancreatic Cancer: A Scoping Review
Source: Cancers (Basel). 2024 Apr 24;16(9):1632. doi: 10.3390/cancers16091632 (PMC11083108; doi:10.3390/cancers16091632)
Supplement: Supplementary file 1 [file cancers-16-01632-s001.zip › cancers-2909840/Supplementary Figure 1.pptx]

## Slide 1
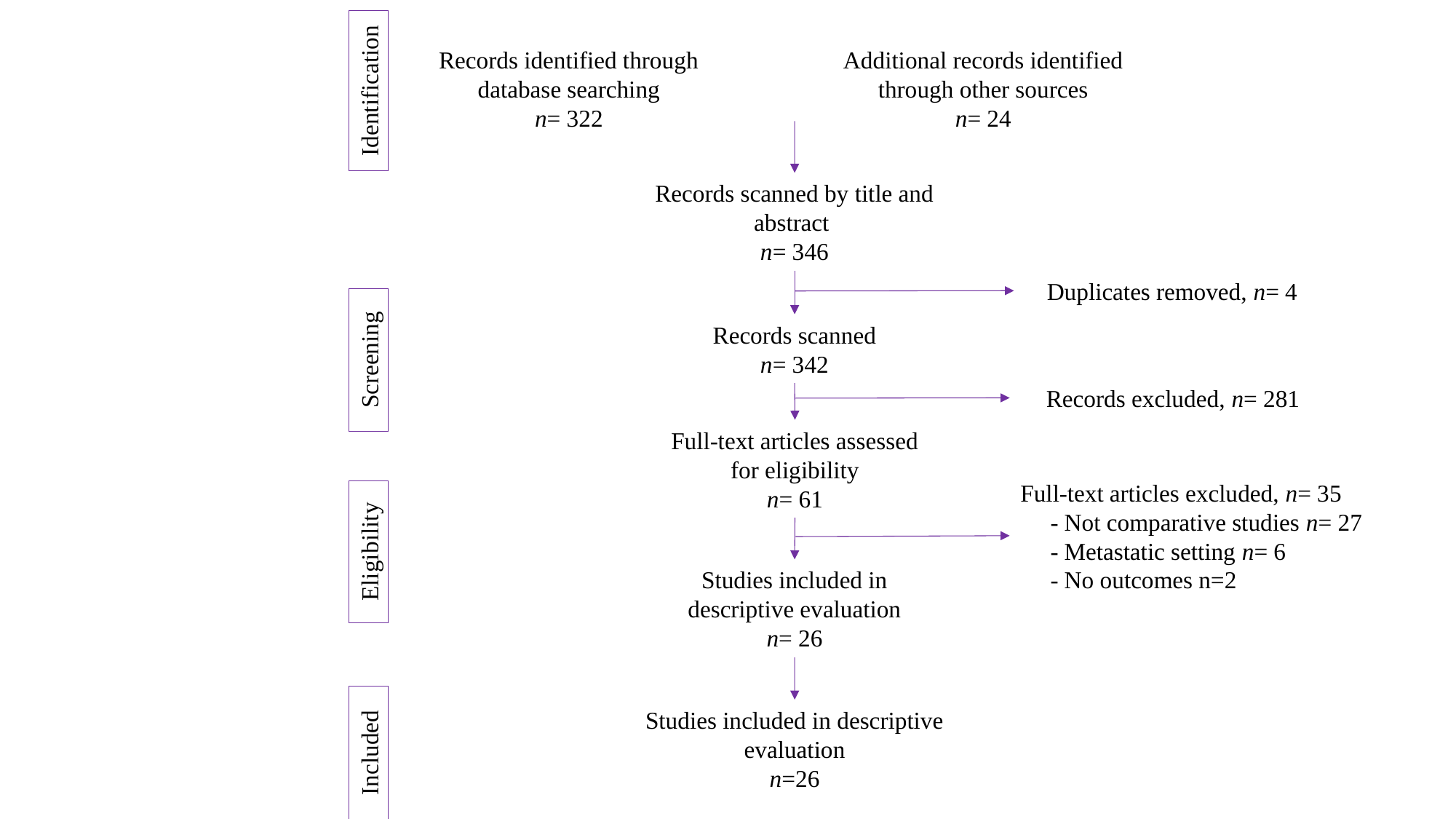

Records identified through database searching
n= 322
Additional records identified through other sources
n= 24
Identification
Records scanned by title and abstract
n= 346
Duplicates removed, n= 4
Records scanned
n= 342
Screening
Records excluded, n= 281
Full-text articles assessed for eligibility
n= 61
Full-text articles excluded, n= 35
　- Not comparative studies n= 27
　- Metastatic setting n= 6
　- No outcomes n=2
Eligibility
Studies included in descriptive evaluation
n= 26
Studies included in descriptive evaluation
n=26
Included
