# Supplementary material for: Evidence and Future Perspectives for Neoadjuvant Therapy for Resectable and Borderline Resectable Pancreatic Cancer: A Scoping Review
Source: Cancers (Basel). 2024 Apr 24;16(9):1632. doi: 10.3390/cancers16091632 (PMC11083108; doi:10.3390/cancers16091632)
Supplement: Supplementary file 1 [file cancers-16-01632-s001.zip › cancers-2909840/Supplementary Figure 2.pptx]

## Slide 1
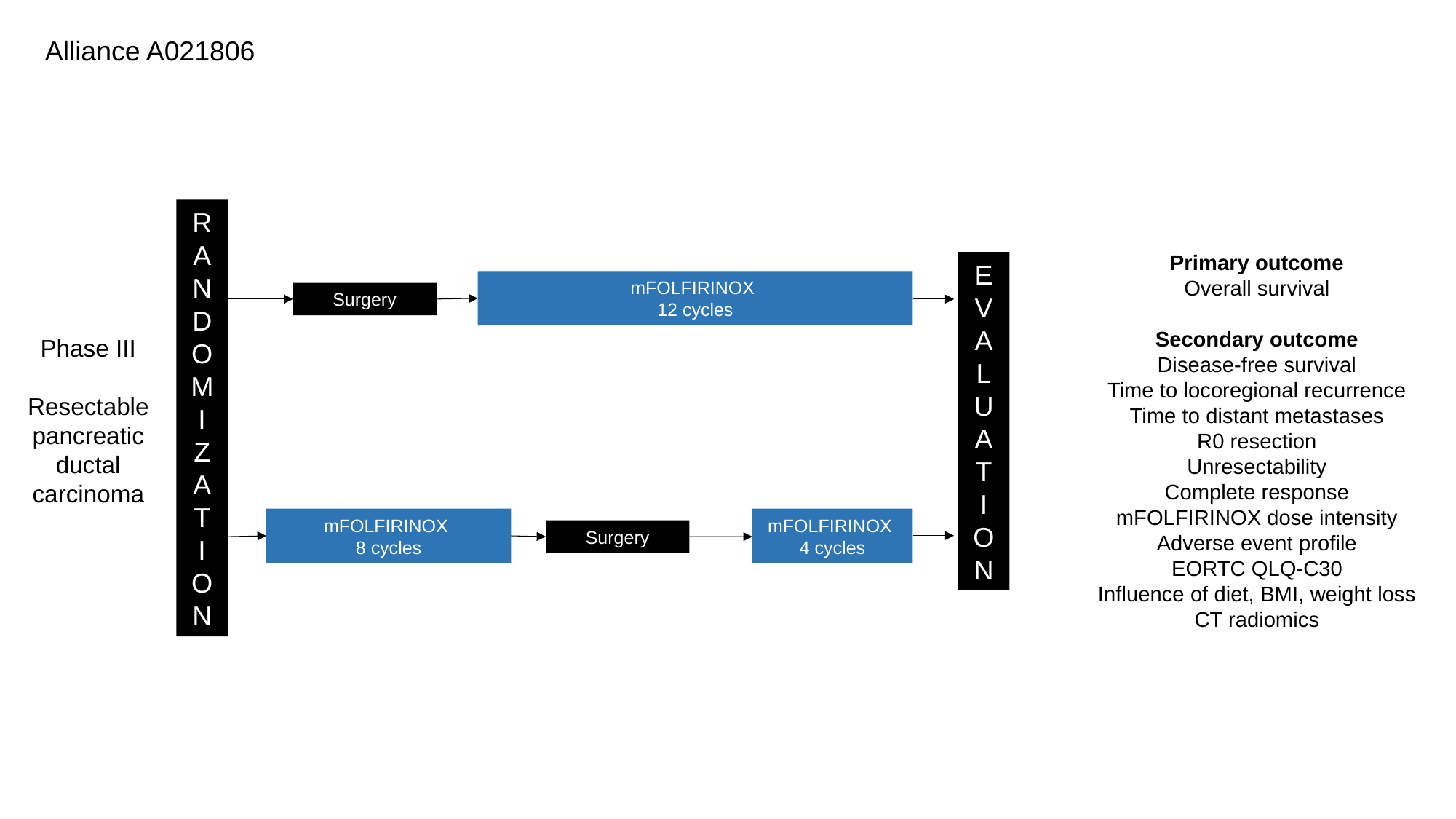

Alliance A021806
R
A
N
D
O
M
I
Z
A
T
I
O
N
Primary outcome
Overall survival
Secondary outcome
Disease-free survival
Time to locoregional recurrence
Time to distant metastases
R0 resection
Unresectability
Complete response
mFOLFIRINOX dose intensity
Adverse event profile
EORTC QLQ-C30
Influence of diet, BMI, weight loss
CT radiomics
E
V
A
L
U
A
T
I
O
N
Surgery
mFOLFIRINOX
12 cycles
Phase III
Resectable pancreatic ductal carcinoma
mFOLFIRINOX
8 cycles
Surgery
mFOLFIRINOX
4 cycles

## Slide 2
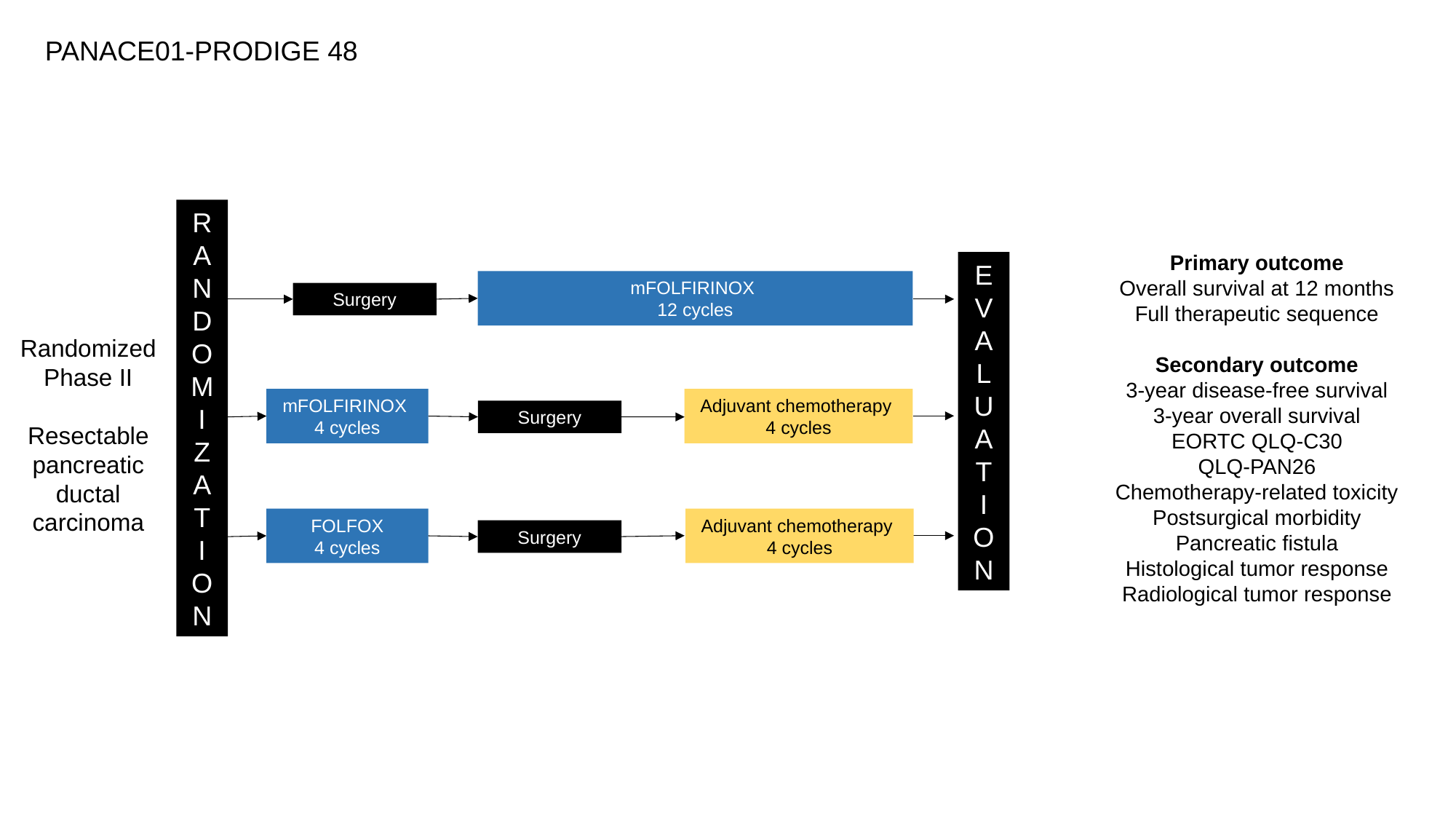

PANACE01-PRODIGE 48
R
A
N
D
O
M
I
Z
A
T
I
O
N
Primary outcome
Overall survival at 12 months
Full therapeutic sequence
Secondary outcome
3-year disease-free survival
3-year overall survival
EORTC QLQ-C30
QLQ-PAN26
Chemotherapy-related toxicity
Postsurgical morbidity
Pancreatic fistula
Histological tumor response
Radiological tumor response
E
V
A
L
U
A
T
I
O
N
Surgery
mFOLFIRINOX
12 cycles
Randomized
Phase II
Resectable pancreatic ductal carcinoma
mFOLFIRINOX
4 cycles
Surgery
Adjuvant chemotherapy
4 cycles
FOLFOX
4 cycles
Surgery
Adjuvant chemotherapy
4 cycles
